# Using the Theoretical Framework of Acceptability for qualitative assessment of the "COMBAT" VAW intervention in Ghana

**DOI:** 10.1371/journal.pgph.0000269

**Published:** 2022-05-02

**Authors:** Ruby Hornuvo, Adolphina Addo-Lartey, Deda Ogum Alangea, Phyllis Dako-Gyeke

**Affiliations:** 1 Department of Social and Behavioural Sciences, School of Public Health, University of Ghana, Accra, Ghana; 2 Department of Epidemiology and Disease Control, School of Public Health, University of Ghana, Accra, Ghana; 3 Department of Population, Family, and Reproductive Health School of Public Health, University of Ghana, Accra, Ghana; University of the Witwatersrand, SOUTH AFRICA

## Abstract

Violence against women (VAW) is a global health problem, which leads to negative sexual, reproductive, mental and physical health outcomes, as well as death in extreme cases. Globally, over 35% of women are reported to have experienced some form of violence, while in Ghana, 37% of women have ever experienced physical violence. Considering that the sustainability of community-based VAW interventions is largely dependent on ownership, this study used the Theoretical Framework of Acceptability (TFA) to assess the COMBAT (Community Based Action Teams) intervention (for example, ethicality, self-efficacy, and intervention coherence) which was implemented to prevent violence against women in Ghana. This qualitative research study was conducted at baseline and end-line of the intervention, which used COMBAT as a vehicle to change social norms on gender and violence in the Central region, Ghana. Participants comprised of adult (women and men) who reside in the Agona District. We analyzed the transcripts from eight (8) Focus Group Discussions conducted within one of the two intervention recipient sites (4 female community FGDs and 4 male community FGDs). Data was analyzed thematically using the Nvivo software version 12. Through the application of the TFA, the findings show that community members perceive VAW as inappropriate at the individual and community levels (ethicality), have good knowledge of the COMBAT intervention and how it works (intervention coherence). Also, the study shows that participants have a positive attitude towards the intervention and its components (positive affective attitude), and could take up components of the intervention, by reporting and seeking for VAW support (positive self-efficacy). There was a perceived reduction in VAW practices in the various communities, as well as a reduction in the abuse of women, thereby improving their well-being (intervention effectiveness). The COMBAT strategy was accepted by the community members hence could be sustained as a culturally appropriate intervention for preventing VAW.

## Introduction

Violence against Women (VAW) is recognized globally as a public health problem, which is defined as, “acts, or threats of acts intended to hurt or make women suffer physically (such as, beating, biting, kicking, slapping, strangling, and having a knife or gun used on the woman), sexually (for example, forced sex, forced involvement in degrading sexual acts, denial of contraceptive use or right to protect against sexually transmitted infections) or psychologically (for example, constant belittlement or humiliation, financial restrictions and other forms of controlling behaviours)” [1–4]. Globally, over 35% of women are reported to have ever experienced either intimate partner violence or non-intimate partner violence [5,6]. The prevalence of violence against women showed that the Africa region recorded highest rates for both IPV (36.6%) and non-intimate partner violence (11.9%) [7]. In Ghana, it was reported that 92% and 34% of women experience sexual and psychological violence from their partners, respectively [8].

General risk factors for experiencing VAW include a history of, or exposure to violence during childhood, witnessing family violence, alcohol and drug use, stress, antisocial personality disorder, communication challenges between partners, and having multiple partners or suspicion of such [5,6]. Depression, disability, and male partner control were also found to be associated with women’s exposure to violence [9]. Relatedly, VAW experiences are known to expose women to suicidal behaviours, Sexually Transmitted Infections (STIs), physical injuries, induced abortion, and low birth weight or premature birth of babies [7]. Furthermore, in Tanzania violence against girls in schools (30%) is identified as a major contributing factor toward high school dropout rates among girls [10]. The entrenched nature of violence perpetrated against women is of public health concern, considering that VAW is also known as a leading cause of homicide among women globally [1].

Unfortunately, Violence Against Women and Girls (VAWG) is still socially tolerated and justified in many African communities, where these acts are deeply rooted in systemic gendered inequalities, which manifest at the individual, interpersonal, community, and societal levels [10]. For instance, in Ethiopia, two of every three women (68%) and one of every two men (45%) believe that wife beating is justified under specific circumstances [10]. Such acts may be shaped by law, policy, social norms and power relations which contribute to the public’s understanding of VAW occurrences. Similarly, earlier studies have established that VAW in Ghana may result from expectations of existing dominant patriarchal norms, entrenched by religious beliefs and socio-economic status, which enforce inflexible gender hierarchy [11]. Thus, women are expected to be submissive to their male partners and demonstrate unquestioning respect, be dutiful, and serviceable, to the extent that going against or challenging abuse may be interpreted as an attempt to disrupt that authority [11].

Over the years, various forms of interventions for addressing VAWG have been developed [12]. These are used as strategies to influence policies, strengthen health systems and political structures to effectively and efficiently respond to VAW as a public health problem [12]. Interventions including research, advocacy, resolutions, frameworks, and sensitization are often executed to control VAW, globally [12–14]. These include the multi-country study conducted by the World Health Organization (WHO) which contributed toward increased awareness among women and VAW victims and led to national and educational policy reforms [15,16]. Also, there is the United Nations (UN) resolution which sought to eradicate all forms of violence against women, and the development of the RESPECT Women Framework by the WHO, based on the UN framework for action to prevent VAW [16]. This framework was designed for policymakers and comprises seven (7) strategies that could be implemented in designing, planning, implementing, monitoring and evaluating interventions for preventing VAW [16].

Additionally, community-based interventions have been executed across sub-Saharan African countries to control VAW in communities. Such interventions draw on the conceptual meanings of “community” as the setting (i.e. geographical location) where the interventions are implemented, and or the target of change, which broad systemic changes in public policy for community-wide institutions and services may focus on [17]. McLeroy et al. (2003) further considers the possible emphasis on the community not only as a resource, but also as an agent. The later interpretation is driven by the belief that high degree of community ownership and participation is essential for sustaining interventions, while reinforcing the natural adaptive, supportive and developmental capacities of communities [17]. Examples of community-based VAW interventions include the Micro-finance for AIDs and Gender Equity (IMAGE), implemented in rural South Africa. The project provided credit and savings assistance to poor rural women to be used for income-earning jobs aimed at contributing towards their empowerment, self-esteem and self-confidence, as a strategy for reducing IPV [18]. This VAW project further influenced the Microfinance and gender training to reduce violence against women **(**MAISHA) intervention, which was implemented in Tanzania [19]. In addition, the SASA (Start-Awareness-Support-Action) intervention, which was conducted in Kampala, Uganda, assessed the impact of community mobilization toward preventing violence against women and reducing risk of HIV [20].

Similarly, in Ghana, the Rural Response System (RRS) model, a community-based intervention developed by the Gender Studies and Human Rights Documentation Centre (Gender Centre) in 2002, seeks to tackle violence against women and children in Ghana [21,22]. The Community Based Action Teams (COMBAT) programme uses a community response model (Rural Response System) which recruits and adopts a curriculum to train team members. Following which, COMBAT members engage in sensitization and awareness raising activities within communities, case management, counselling, mediation as well as act as referrals between state agencies and VAW victims. These activities aim to: (i) raise public awareness about the causes and consequences of VAWG and women’s rights, and change harmful social norms and practices that perpetuate violence; (ii) enhance coordinated efforts between community members, state agencies and other key actors to respond to violence within communities; and (iii) establish referral systems for survivors of violence to access support services [8,22]. The primary outcome of this intervention is a reduced incidence of violence against women, which will ultimately improve their wellbeing. Considering that the sustainability of this intervention is largely dependent on community ownership, the Theoretical Framework of Acceptability (TFA) was used to assess the intervention. This study explored community members’ knowledge, perceptions, and acceptance of the COMBAT intervention, which were used as basis for assessing community acceptability of the COMBAT.

## Materials and methods

### Study design

We analyzed this qualitative research component from the larger community-based intervention trial, which assessed the community-level impact of the Rural Response System that uses Community-Based Action Team ‘COMBAT’ for preventing VAW in Ghana [22]. The qualitative data was used to assess the acceptability of the COMBAT intervention with the application of the Theoretical Framework of Acceptability.

#### The Theoretical Framework of Acceptability (TFA)

The TFA developed by Sekhon et al consists of conceptually different constructs (affective attitude, burden, ethicality, intervention coherence, opportunity costs, perceived effectiveness and self-efficacy) that capture important dimensions of acceptability of health interventions [23]. The TFA is appropriate because its constructs are relevant for assessing interventions at the individual, interpersonal and community levels. Relatedly, the ‘COMBAT’ intervention draws from community leadership and ownership, as well as trust for trained local actors. Therefore, its assessment requires use of a rigorous framework whose elements would be applicable for assessing how context specific needs are addressed through the delivery of an acceptable and culturally appropriate intervention. In this case, acceptability is defined as the perception among beneficiaries (for example, community members) that a given treatment, service, practice or innovation (such as COMBAT activities) is agreeable, palatable, or satisfactory. This reflects a more collectively shared judgement about the nature of the COMBAT intervention [23,24].

### Study setting

The setting for this study was the Agona district located in the Central region of Ghana. This district was part of the four (4) districts selected to participate in the trial conducted by the Ghana COMBAT Health Promotion Study (Ghana CHiPS project) which lasted for three years and included a baseline survey, midpoint and end-line evaluation [8,22].

The selection of the districts was done using a survey map of the Central region, which had inland and coastal districts. After eliminating some districts (Assin North, Assin South, Ajumako, Enyan and Essiam) due to previous intervention work on Gender-Based Violence (GBV) that was carried out in those districts, two (2) inland and two (2) coastal districts were then purposively selected as study sites. These districts were then grouped into intervention arms (Agona,Komenda/Edina/Eguafo/Abriem) and control arms (Upper Denkyira, Abura/Asebu/Kwamankese) [22]. Forty (40) localities, (ten (10) per district) were randomly selected using a list acquired from the Ghana Statistical Service (GSS).

The Agona district, located in the Central region of Ghana is sub-divided into Agona East and Agona West with district capitals of Agona Nsaba and Agona Swedru, respectively. It has a total population of 201,278 with 50,499 households. The Agona district is characterized mainly by agricultural activities; farming, skilled agricultural, fishery and forestry workers [25,26].

### Selection and description of participants

A total of eight (8) community FGDs were conducted in the Agona District of Ghana and comprised 70 participants from both baseline and the end-line components of the study. That is, two (2) baseline female community FGDs (n = 19), 2 baseline male community FGDs (n = 17), 2 end-line female community FGDs (n = 17) and 2 end-line male community FGDs (n = 17).

Participants for all community focus group discussions were purposively selected to include those who have been reached by COMBAT members as well as others who have not. These could be community members who may have had prior VAW experiences, or not. Participants comprised all adult (women and men) who reside in the Agona District (comprising of 10 communities) in the Central region of Ghana. Men were included because; this is a community-owned intervention, geared towards community involvement.

### Data collection techniques and tools

Focus Group Discussions (FGDs) were used to provide an understanding of the influences of the COMBAT intervention if any, at the individual, community and institutional levels. FGD guides were developed based on the primary and secondary outcomes of the RRS trial and translated into local dialects (Fante and Twi) by an independent consultant and then edited by bi-lingual members of the project team at the University of Ghana. The revised translations were independently back-translated by another consultant who had not seen the English guides. The project team then used a consensus-building translation approach to finalize the translated interview guides.

Each FGD comprised 7–10 participants, led by a trained facilitator, supported by a note-taker and averagely lasted for 1-hour 45 minutes. Discussions were organized at private locations away from the community centres. Informed consent was sought and FGDs were audio-recorded. FGDs were organized differently for women and men to permit for the expression of gendered opinions within a relaxed and convenient atmosphere without any apprehension. Also, they were gender-matched, where male research assistants moderate male FGDs and female research assistants moderate female FGDs. This was conducted at baseline and end-line to enable assessment of improvement in knowledge and understanding as well as to qualitatively evaluate the intervention delivery and outcomes.

### Data processing and analysis

All recorded FGDs were transcribed verbatim and augmented with the researchers’ field notes taken through observation. A thorough re-reading of the transcripts and notes was done and this aided in the development of the initial codebook This was done by two (2) lead researchers, whilst coding was done by four (4) research assistants. The coding process was interspersed with meetings between the research assistants and researchers, during which the identification of new codes was discussed, decisions on inclusion of emerging codes taken, and codebook refined and finalized. Thematic analysis, employing both deductive and inductive processes was used in analysing the data. All transcripts were imported into QSR NVivo 12 software for analysis. The data was explored by first running queries. The initial codes generated based on the TFA constructs were used to code (generate nodes), as each transcript and FGD guide was read line-by-line and relevant information based on TFA constructs were dropped into created nodes. The coding was then reviewed where some nodes were re-arranged and others merged to develop themes and sub-themes. The codebook initially developed was revised throughout the coding process. The coded themes and sub-themes were then exported from NVivo into Microsoft word for further reviewing and interpretation of data. Identified themes were reviewed by the two lead researchers to ensure they align with the coded extracts and the whole data set. Following which, themes were then verified as major categories were compared with each other and consolidated to avoid overlaps. During this process, the most vivid and compelling extracts were selected, connecting the analysis to the research objective, which are presented in the result section of the work.

### Ethical considerations

Due to the sensitive nature of the trial, the study was designed to prevent participants exposure to further risks with the privacy and security of participants and researchers ensured. All project staff were trained on gender, gender-based violence and research ethics prior to the implementation of the trial. All prospective participants were informed about the purpose, risk, and benefits and that participation in the trial was voluntary and appropriate informed consent was provided, in participants’ language of preference (English or local dialects). They were also informed that they may withdraw at any stage or skip any question in the research with no adverse consequences to them. Participants’ information was handled confidentially. All participants’ information was saved in folders on a password-locked computer that only investigators had access to, and findings were reported with complete anonymity. A written informed consent (this was achieved via thump-printing for illiterate participants) was sought from all participants before participating in this study.

Also, provision was made for interviewers to refer participants to state agencies such as the Domestic Violence and Victim Support Unit (DOVVSU) and the Social Welfare whenever they encounter situations where participants demonstrated distress or report being emotionally impacted by the research questions or intervention.

Ethical approval for the study was secured from the Institutional Review Board at the Noguchi Memorial Institute for Medical Research, University of Ghana, (CPN-006/15-16) and the South African Medical Research Council’s Ethics Committee (EC031-9/2015).

The authors constitute a team of African women researchers, who are located within both academia and practice on the continent. These researchers evaluated the intervention (baseline, mid-line and end-line). They however, conducted fieldwork alongside the organization that implemented the intervention. The last author, PDG was the lead person who conceptualized and initiated the writing of this paper, PDG refocused this position on herself. She is a Ghanaian who was born in Ghana and has lived within this context for the most part of her life as a lecturer and a researcher, within academia. She has no personal experience of gender-based violence, she has however often witnessed it in the most diverse forms in Ghana and elsewhere. With full knowledge that these previous observations may bias her perspective on this matter, a larger team of experts with relevant skills in qualitative research were involved, who were a part of this study from the data collection through to the analysis and presentation.

## Results

The findings of the study are presented under the ensuing headings; Socio-demographic characteristics of participants and acceptability of the COMBAT intervention using the TFA constructs which includes: self-efficacy, ethicality, affective attitude, intervention coherence and perceived effectiveness.

### Socio-demographic characteristics of participants

A total of seventy (70) participants were involved in the baseline and end-line community FGDs, comprised of 36 female participants, with age ranged between 22 and 66years (Table 1). Participants were mostly from Obratwawu, Nyamendam, Kwame Kwei, Kwadwo Ashong, Otsenkorang, Edukrom, Nsuansa and Kwame Adwe communities in the Agona district and are mostly farmers with few having formal employment. The longest years of length of stay in the community recorded among participants was 61 years (Table 1).

**Table 1:** Socio-demographic characteristics of baseline and end-line participants.

Characteristic of Participants	Number of Participants		
	Baseline Participants	End-line Participants	Total
Obratwawu	10	9	19
Nyamendam	3	-	3
Kwame Kwei	3	8	11
Kwadwo Ashong	1	5	6
Otsenkorang	8	2	10
Edukrom	8	6	14
Nsuansa	3	3	6
Kwame Adwe	-	1	1
**Total**	**36**	**34**	**70**
**Sex**			
Female	19	17	36
Male	17	17	34
**Total**	**36**	**34**	**70**
**Age**			
**<**35years	20	16	36
>35years	16	18	34
**Total**	**36**	**34**	**70**
**Educational Level**			
No formal Education	4	3	7
Primary	2	4	6
JHS/SHS/Secondary	23	23	46
‘O’ Level/Middle School	4	3	7
Tertiary	3	1	4
**Total**	**36**	**34**	**70**
**Ethnicity**			
Akan (Fante, Gomoa, Wassa)	34	33	67
Ewe	2	1	3
**Total**	**36**	**34**	**70**
**Religion**			
Christianity	31	28	59
Islam	5	3	8
Traditional	-	1	1
None	-	2	2
**Total**	**36**	**34**	**70**
**Occupation**			
Unemployed	1	1	2
Food vendor/Petty trading	9	6	15
Artisans	5	6	11
Poultry Farmer/Fisherman/Farmer	17	17	34
Formal Employment	2	4	6
Retired/Pensioner	2	-	2
**Total**	**36**	**34**	**70**
**Marital Status**			
Single	3	7	10
Co-habiting	8	5	13
Married	22	21	43
Divorced/Widowed/Separated	3	1	4
**Total**	**36**	**34**	**70**
**Length of Stay in Community**			
<20years	15	15	30
20-29years	9	13	22
30-39years	4	4	8
40+years	8	2	10
**Total**	**36**	**34**	**70**

### Self-efficacy

Self-efficacy as an acceptability construct explored community members’ confidence that they can perform the behaviour necessary to partake in the COMBAT intervention. This included their ability to take up components (e.g., identification of violence against women, ability to report, etc.) of the COMBAT intervention and seek support.

The study shows that several participants were able to understand the components of the intervention and expressed their ability to transfer such knowledge to others. Participants believe that this action of transferring what they know to others can help promote peace as well as ensure the sustainability of the intervention. For instance, a participant mentioned:

*We can become ambassadors for peace. We can and will educate other friends and relatives. We will also impart it to our children. For the children to also learn from their parents. **(R5, Male, Nyamendam).***

*We would relay this information to our children in the future which would serve as a guide for them hence the combat work would not be a thing of the past **(R4, Female, Edukrom).***

Also, female participants reported to have become enlightened due to the awareness creation by the COMBAT members and as such, they can take actions to prevent themselves from being victims of violence against women as expressed using the following quotes:

*Now I have learnt how to deal with my anger/temper because our inability to manage our anger well can bring violence **(R5, Female, Nsuansa).***

*Now I know that I need to teach my children especially, the teenagers so that they can protect themselves from getting pregnant at early stages **(R7, female, Edukrom).***

The ability to report violence against women acts or seek support was also realized from the study. Participants indicated that they could seek support from COMBAT members as well as report VAW acts if they experience any form of violence or encounter any form of misunderstanding with their partners. This was evident in one participant’s statement that:

*… if one is experiencing violence*, *it will be prudent for her to seek help from combat and everything will fall in place* ***(R7*, *Female*, *Edukrom***).

Similarly, another participant stated that

*We can invite them to households. We also do report a husband or wife who perpetrates violence **(R2, Male, Obratwawu).***

### Ethicality

This theme explored the community’s view of the COMBAT intervention regarding its appropriateness with their value system. It sought to know if indeed condemning VAW as a core element of the COMBAT intervention was good fit with their cultural value system. Findings showed participants stating emphatically their non-acceptance of VAW as expressed by one participant that *‘I do not tolerate violence against women’* ***(R9*, *Male*, *Kwame Kwei)*.** Health issues, specifically psychological consequences, physical injuries or death in extreme cases were indicated as some reasons why VAW is not appropriate. This is shown in the quote below:

*I don’t tolerate violence against women. This is because violence against women can either damage the brain or bring about deadly sickness. Some violence can even result to death. A stroke or punch can make someone unconscious. This can lead to death. Most physical assault injures the victim who are often women. Some men do not know how to beat women lightly. Small beatings result into injury, which can lead to death. There some parts of the woman’s body which needs not to hit. Hitting those parts of the woman’s body can make the woman unconscious. **(R7, Male, Nyamendam).***

Also, religious belief was mentioned as a reason why some participants do not tolerate VAW because their faith does not tolerate violent acts against women. This was evident in a participant statement below:

*The main reason why I don’t tolerate violence is that it is written in the bible “do not extend your anger to another day.” Do not hold anger for a very long time. If you do hold anger or grudge against someone for a very long time, it culminates in evil thought. This will lead you to undertake an action that brings regrets later in life **(R1, Male, Obratwawu**).*

The consequences of VAW on the life of children was also indicated as a reason why VAW is not appropriate. The fear that children may suffer the psychological consequences of observing violent acts and this might translate into their adult lives, makes VAW unacceptable for some participants. This was stated by one participant:

*I don’t tolerate violence against women because it does not bring peace to the home. It doesn’t make peace to prevail in the house. It also affects children psychologically. If violence occurs, children who find themselves in the marriage become confused. They wouldn’t know which sides to take. The children become miserable in life. **(R6, Male, Nyamendam)***

### Affective attitude

This theme explores how community members feel about the COMBAT intervention, and their positive or negative description of the experienced intervention. A positive attitude towards COMBAT intervention was realized among many participants. Some participants expressed the benefits they have derived from their exposure to the COMBAT intervention at the community level, as well as at individual levels. Community-level benefits included reduction of VAW practices, peace and enlightenment, as shown below:

*COMBAT has really helped us, they have helped reduced violence against women in our community, if I compare the situation before and after COMBAT intervention, there have been changes, positive changes because we have all learn something from COMBAT, and we are now enjoying peace in our communities. **(R5, Female, Nsuansa).***

Likewise, individual-level benefits included positive behavioural changes and enlightenment, which has helped contribute to peace. For instance, a participant stated that:

*… some of us were impatience about certain things, we use to disrespect our husbands, but we have been taught that exchanging words with your husband is not the best, those who accepted the teachings have benefited. Now there is peace in our households and in our community. I do not know about others, but for me, I have learnt a lot **(R4, Female, Nsuansa)***.

Furthermore, participants expressed attitudes such as admiration regarding the approach used by COMBAT in the delivering of the intervention components. This was expressed by some participants:

*…. The ways and mechanisms used during discussions and interactions has just been beyond my mind. Therefore, I feel so happy within me when I see you people… **(R7, Male, Obratwawu).***

Whilst another stated that:

*We have some kind of trust in them that they will always be there to help us solve our marital issues, and because of that trust we have in them, they will always be our first point of call anytime we experience violence. **(R1, Female, Otsenkorang).***

The community members also appreciated the COMBAT intervention. This was conveyed by one participant that:

*To be very honest on behalf of the whole community, I will say a big thank you to the COMBAT team simply because they have done a good job. Whenever teachings or education they brought to us, we have indeed benefitted from it. **(R8, Male, Kwame Kwei).***

In this regard, they expressed their desire for the intervention to be sustained in the community:

*But human beings are just like pomade, we melted the moment the COMBAT people took over with their teachings and education…COMBAT has really helped a lot, therefore, we say thank you very much and they shouldn’t stop the work they are doing…so he pleads with whoever brought the idea to still continue and may God also bless them **(R4, Male, Obratwawu).***

### Intervention coherence

This construct explores participants’ understanding of the COMBAT intervention and how it works. Findings from the study revealed that participants have good knowledge of the COMBAT intervention and how it works, as well as its components. Community members acknowledged the fact that they were engaged by the Gender Center to select representatives from the community to serve as COMBAT members; hence, COMBAT members were selected from the community:

*Gender Center came here some time ago, they asked us to select some of our members to go for training so that they can also come and teach us. So, COMBAT members are people from our own communities **(R6, Female, Edukrom)***.

In addition to the above, participants also expressed their knowledge of the fact that COMBAT members are responsible for awareness creation on VAW and a relationship based on equality as well as resolve or mediate VAW issues. This was expressed by one participant in the statement below:

*COMBAT are trained to provide information on violence against women. As a result of the information provided by COMBAT, there has been a decrease of reported violence against women cases in the community **(R3, Male, Obratwawu).***

Similarly, on relationship based on equality, one participant expressed that:

*It was about my marriage issues and also they taught me about how men should help their wives in the household. When you also have issues, they can help you fix it **(R3, Male, Obratwawu).***

Community members also indicated that, COMBAT act as referrals to formal VAW support agencies such as the Police, DOVVSU and the Social Welfare Services:

*… The COMBAT members have also been trained to refer violence cases to state institutions. As a matter of fact, it is the responsibilities of COMBAT members to refer cases to state institutions **(R4, Male, Nyamendam)***.

The above-mentioned are all important components of the COMBAT intervention. It is however interesting to note that, male participants seem to have a comprehensive knowledge of the COMBAT intervention and its components as compared to female participants. For instance, males could mention the roles of COMBAT, which includes awareness creation, provision of support, mediation and referrals to state agencies. These are expressed in some quotes below:

*What they made us to understand is that the COMBAT members are volunteers who mediate violence cases between a man and a woman. And that they should refer any case that surpasses their capacity to state institutions. It is the responsibility of COMBAT to mediate violence cases such as a man perpetrating physical violence or a man refusing to accept his responsibilities **(R6, Male, Obratwawuo).***

*COMBAT are people who have been trained to provide information in household on violence against women. So that men and women can live in harmony. They are also trained on the laws of Ghana. This is to enable them share their knowledge with the community members **(R4, Male, Obratwawuo).***

Unlike males, the female participants mostly knew about awareness creation and mediation as roles of COMBAT:

*…we were told that some people had undergone training are going share what they went to study with us. There, they mentioned their name as combat members who will assist in resolving misunderstanding and mediating marital issues as well as settling issues that are not going well at home **(R5, Female, Edukrom**)*

*They are our own community members who were selected by the government to go for training on violence, and come and educate us so we can help prevent violence **(R2, Female, Otsenkorang**)*

### Perceived effectiveness of the COMBAT intervention

This construct explored the level to which the COMBAT intervention has attained its purpose, thus reduced VAW, improved upon the health of women and reduced the victimization of women. It was realized from the study that, even though VAW practices have not stopped completely, there has been a perceived reduction of VAW practices in the various communities of the Agona District. For instance, some stated that:

*Formerly, Nyamedam use to record high violence cases. Education given by COMBAT during funerals has really helped. It has contributed massively towards the reduction of violence in this community. **(R8, Male, Nyamendam).***

Another participant also indicated that, in addition to the reduction in VAW acts, there has been peace in the community due to the COMBAT intervention:

*COMBAT has really helped us, they have helped reduced Violence against women in our community, if I compare the situation before and after COMBAT intervention, there have been changes, positive changes because we have all learn something from COMBAT, and we are now enjoying peace in our communities **(R5, Female, Nsuansa).***

The study further revealed that COMBAT intervention has contributed to a reduction in the abuse of women as expressed in the quotes below:

*Before the COMBAT intervention, some people did not know that it was wrong to beat their wives, but now they know beating a woman is a criminal offence, so they have stopped. **(R8, Female, Otsenkorang).***

*I fully accept the support provided by COMBAT. If care is not taken, the community will start recording violence against women related death. The intervention by COMBAT has reduced violence against women **(R1, Male, Obratwawuo**)*

*Some women get beaten even when they haven’t done anything wrong but now all has ceased **(R2, Female, Otsenkorang)***

It was also revealed that, the introduction of the COMBAT intervention has led to changes in gender roles as indicated below:

*The support COMBAT brought to us is that even if the woman is pregnant, she will pound fufu for her husband, at the same time making soup but since COMBAT came it has change the attitudes of men, now when the woman is cooking the man can also help out **(R9, Male, Kwame Kwei).***

*The combat work has really helped us. This is because my husband will not even lend a helping hand when I am doing some chores at home like to fetch water before he goes to the farm but now, he does while I do other chores at home (**R7, Female, Edukrom**)*

*It has really been beneficial because the men now help their wives in the kitchen and the pressure of they doing double and stressful chores at home has changed (**R6, Male, Obratwawu**)*

## Discussion

This paper assessed the acceptability of the COMBAT Intervention employed for preventing VAW in the Central region of Ghana using the Theoretical Framework of Acceptability (TFA). To the best of the researchers’ knowledge, this is the first study to assess the acceptability of a community-based VAW intervention with the application of the TFA. Application of the TFA constructs provided a more diverse assessment of the intervention acceptability compared to when assessing intervention acceptability in general. It offers a “*multi-faceted construct that reflects the extent to which people delivering or receiving a healthcare intervention consider it to be appropriate*, *based on anticipated or experienced cognitive and emotional responses to the intervention”* [27,28]. In this regard, community members expressed a high level of self-efficacy in relation to the COMBAT intervention as participants felt confident that they can practice components of the intervention. Male and female community members of Agona district understood the components of the COMBAT intervention, have become aware of their rights and therefore can report acts of violence or seek support. And this self-efficacy is necessary in preventing violence against women, as studies showed that an increase in self-efficacy and awareness helps prevent the act [29]. Therefore it highly recommended for VAW interventions by the World Health Organization [14]. There has also been a positive behavioural change among members of the community, which has contributed to a reduction in VAW cases in the community.

The Ethicality of a community-based intervention is key in its acceptability by the community, as an intervention, which is not culturally or ethically appropriate, will tend to have less participation and engagement from the community members it was intended for. Ethically appropriate interventions are therefore more appreciated and easily sustained by community members. Findings from the trial revealed that the COMBAT intervention was ethically appropriate with the value system of the people of the Agona district in the Central region of Ghana. Community members expressed their non-acceptance of the perpetuation of violence against women stating reasons such as possible negative health consequences (psychological, injuries and death in extreme cases), religion and effects on children. These results further confirm findings from a multi-site study conducted across Africa, Americas, Eastern Mediterranean, Europe, South-East Asia and Western Pacific on the health consequences of VAW [5,7] as well as the community’s views on the effect of VAW on the health of women and children [30,31].

The application of the TFA further revealed a positive affective attitude towards the COMBAT intervention by the community members. These included benefits they have derived from their exposure to the COMBAT intervention both at the community level and at the individual level. Community members are now enlightened on issues related to violence against women and have experienced a reduction in violence against women in the community since the introduction of the COMBAT intervention. Community members additionally expressed their appreciation and support for the COMBAT intervention and their desire for the intervention to be sustained in the community. This is not common with a community-based VAW intervention, which was implemented in Uganda; this study, only sought to assess the impact of the intervention in preventing diverse forms of violence and not how the community feels about the intervention after participating in it [32].

Intervention Coherence, which explores the extent to which participants understand the COMBAT intervention and how it works, consequently influences the ability to participate in the intervention, help eradicate misconceptions about VAW support systems as well as influence behaviours towards the intervention and empower them to prevent VAW. As evident in a review of interventions to prevent violence against women and girls (VAWG), raising awareness and changing social norms are key components in addressing violence against women and girls [33]. The study revealed that community members know who COMBAT members are, their roles and responsibilities, which includes awareness creation on VAW and a relationship based on equality, resolving or mediating VAW issues as well as serving as referrals to formal support systems, which are core components of the COMBAT intervention. This is in line with other community-based intervention findings, which showed that awareness creation and sensitization helped reduced the incidence of IPV [34,35] as well as agrees with WHO recommendations for preventing VAW [36]. Notably, male participants had a comprehensive knowledge of the COMBAT intervention and its components compared to female participants. While this gendered difference may have contributed to changes in inequalities, social norms and reduction in the perpetuation of violence against women in these communities, it may be a reflection of broader gendered inequalities within the study communities which may allow for male exposure to activities/happenings within the communities’ public sphere, as compared to women’s involvement in reproductive roles at the family level.

Perceived effectiveness of the intervention by community members is also key in measuring the acceptability of the COMBAT intervention. Findings from the larger project indicate that the RRS intervention reduced women’s experiences of sexual IPV (from 17.1% to 7.7%), physical IPV (from 16.5% to 8.3%), severe IPV experienced by women (from 21.2% to 11.6%), depression, and partner controlling behaviour in the intervention areas [37]. Through the application of the perceived effectiveness construct, it was realized that, though VAW practices have not stopped completely, there has been a perceived drastic reduction of VAW practices in the various communities. Also, as the focus is on reduction, it can be said that the COMBAT intervention was very effective in reducing VAW in these communities. In addition to the reduction in VAW cases, the study also showed that COMBAT intervention has contributed to a reduction in the abuse of women in these communities and changes in gender roles, which further translates into the improvement of health among women. And these findings are in line with other community-based VAW intervention studies conducted in Sub-Saharan Africa which revealed that community-based VAW interventions (where communities are engaged to own the intervention to improve upon the health in communities) are more effective in reducing VAW practices as compared to interventions that are not community-based [20,38].

### Implications for policy and practice

Overall, we found that, the COMBAT intervention has good fit with the value systems of the community members. The community members have a good knowledge of the COMBAT intervention and how it works and have expressed a positive attitude towards the COMBAT intervention and its components. They could also take up components of the intervention, by reporting and seeking for VAW support. As such, the COMBAT intervention could be sustained as a culturally appropriate intervention for preventing VAWG in Ghana. Also, policy-makers and relevant stakeholders need to provide support for the sustainability of the COMBAT intervention. This includes policies, human resources, financial and material resources at various community levels.

### Limitations

This paper illustrates the acceptability of the COMBAT intervention as experienced by community members using the TFA. However, we need to mention that, the trial involved several sensitive topics concerning violence, and questions requiring victims to recount traumatic events, which possibly may have led to re-victimization and the risk of psychological distress. We acknowledge that such a situation may have discouraged participants from sharing full details of their experiences or may not have been in a sound frame of mind for accurate recollection. Secondly, this project was funded and as such the intervention cannot be easily replicated by communities without the availability of external sponsorship. For instance, expertise needed for implementing the intervention (for example, training and human resource) were from external sources. Finally, the timeframe (3years) for the implementation and evaluation of the COMBAT intervention may not have been enough to realize significant changes in social norms. Despite these limitations, the study revealed important findings regarding the level of acceptability of the COMBAT intervention.

## Conclusion

Through the application of the TFA, the findings show that community members generally perceive VAW as inappropriate at the individual and community levels (ethicality) and have a good knowledge of the COMBAT intervention and how it works (Intervention coherence). Participants have a positive attitude towards the COMBAT intervention and its components (positive affective attitude), and could take up components of the intervention, by reporting and seeking for VAW support (positive self-efficacy). The COMBAT strategy was accepted by the community members hence could be sustained as a culturally appropriate intervention for preventing VAW.

## Supporting information

S1 Table Table of themes. (DOCX)

S1 File Study guide. (DOCX)
