# Supplementary material for: Using the Theoretical Framework of Acceptability for qualitative assessment of the "COMBAT" VAW intervention in Ghana
Source: PLOS Glob Public Health. 2022 May 2;2(5):e0000269. doi: 10.1371/journal.pgph.0000269 (PMC10021998; doi:10.1371/journal.pgph.0000269)
Supplement: S1 Table — (DOCX) [file pgph.0000269.s001.docx]

| S/N | Themes | Definition |
| --- | --- | --- |
|  | Ability to report VAW acts or seek support from COMBAT/ take up components of the intervention (Self-efficacy) | Community members’ ability to seek support when faced with violence/take up component of the intervention |
|  | Non-acceptance of VAW (Ethicality) | Community members view of VAW acts as wrong and ethically inappropriate |
|  | Positive attitude towards COMBAT intervention (Affective attitude) | Community members’ positive feeling about the COMBAT intervention |
|  | Knowledge of COMBAT intervention and its component (Intervention coherent) | Community members understanding and awareness of the COMBAT intervention (Community-owned initiative, sensitization on violence and gender related topics etc.) |
|  | Reduction of VAW practices in the community (effectiveness of COMBAT intervention) | Community member’s knowledge of reduction in VAW activities in the community |
|  | Reduction of victimization of women (Effectiveness of COMBAT intervention) | Community member’s knowledge of reduction in the victimization of women in the community |

**Table 2: Table of Themes**
