# Supplementary material for: Using the Theoretical Framework of Acceptability for qualitative assessment of the "COMBAT" VAW intervention in Ghana
Source: PLOS Glob Public Health. 2022 May 2;2(5):e0000269. doi: 10.1371/journal.pgph.0000269 (PMC10021998; doi:10.1371/journal.pgph.0000269)
Supplement: S1 File — (DOCX) [file pgph.0000269.s002.docx]

**Focus Group Discussion Guide for Community Members**

**Focus group discussion facilitator:** ______________________

**Notetaker (if applicable):** ______________________________

**Town/community/District:** ________________________

**Date:**_______________________

**Time discussion started:** __________  **Time ended:**________________

**# of Women/Men Participating in Focus Group:**_____________

**INTRODUCE MODERATOR, NOTE-TAKER:**

**INTRODUCE THE PURPOSE OF THE GROUP:**

Let’s go ahead and get started. My name is _____________________ and this is my colleague ____________________. We work for _____________________________. We were here a few months ago to have a conversation with you. Today we are back to continue with the conversation. We are very interested in learning about VAW in this community. We will only share the information we learn today in a general way and in a way that does not reveal the identity of anyone in the group. It’s important that the information shared in this group does not leave this group, so we ask everyone to not share who was here or what was said in the group with others outside the group when you leave here. We really want to hear what you have to say and want you to feel comfortable in answering questions however you want to. There are no right or wrong answers.

________________ is taking notes to make sure that we don’t miss what you have to stay. This will help us later when we go back and organize all the information that was shared today. The group should last for [insert amount of time].

Does anyone have any questions before we begin?

**ICE BREAKER**

Ask participants to wish for something good for their community. Ask why they want that particular wish for the community.

**AWARENESS, PERCEPTIONS, AND PRACTICES OF VAW**

**Now I’m going to ask you a few questions specifically about VAW (Violence Against Women).**

1. What kinds of VAW usually occur in this community? In what situations/circumstances does VAW occur?

2. Personally how do you feel about the occurrence of VAW in this community?

**PROBE:** Is it something that you tolerate/allow? Why? How do you think VAW issues must be addressed in this community?

**VAW INTERVENTION (AWARENESS, KNOWLEDGE & USE)**

4. I would like to know about any kind of information you have received in this community on VAW since our last conversation.

**PROBE:** Has anybody spoken with you about VAW? What kind of information was provided? Who provided you with the information? How often was the information provided? When was the information given to you? What medium was used? How was information conveyed to you?

**PROBE**: Was the information useful? Why? Why not? Was the information acceptable? Why? Why not? How will you use this information in future? Is there any other information you wished to have received? Why? Is there anything you liked/disliked about the information given you?

5. *INTRO: Now I would like to share a fictional VAW story with you and want you to share your opinion about this fictional story.*

Aba is 45 years old and lives in (name town of the FGD). Her husband threatened to hurt her badly if she ever left him. He says: “If you leave, I’ll find you. No matter how long it takes I will look for you. You can’t get away from me. I will destroy your pretty face so no one will ever want you. I will also hurt anyone who tries to get you away from me.” He carries out his threats. For about 2 years now he has beaten her often over every little disagreement between them. She has lost two of her front teeth and has an ugly scar over her left eye. She has lost part of her hair from continuous pulling when he beats her. She walks with a limp because he once dislocated her right knee. One time he beat her so badly she had to wear dark spectacles and cover her face with a scarf for several days. He came with her to the hospital and told the Doctor she had a car accident on her way to the market. The Doctor said nothing. That night he was very nice to her. He cooked dinner, served her and included a bottle of beer. Then he asked her “do you still love me?” she said, “Look at me. My eyes and face are swollen. I can’t even look at myself in the mirror.” He apologized for his behaviour. But that promise barely lasted 48 hours before he was beating her again. What do you think about this problem that 45 year old Aba is facing? (Domains: Mediation, Referrals, and Support).

**PROBES**

5.1 What can Aba do in this situation? 5.2 What choices are available to Aba in this community so that this beating would stop? Can Aba act on these choices? If so how? If not, why not? 5.3 Are there people in the community that Aba can invite to mediate the situation between her and her husband? If so how? Who can she invite? 5.4 What do you think will be the reaction of Aba’s husband when he finds out that someone has been invited to mediate? 5.6 If Aba does not know of community members who can help in mediation can you help her contact people in the community for help? Who are they? How did you get to know about them?

6. Now I would like to talk about another scenario. So Aba eventually confides in a community member for assistance. The community member realized the situation was bad so he referred Aba to various places she can go and receive support.

**PROBES**

**6.1** Who will this community member that Aba confides in be? Why do you say that?

6.2 Where do you think Aba will be referred to receive assistance by this community member? (Professional, financial, physical, social, state services, political, media, etc.)

6.3. What kinds of support can Aba receive from the person she confides in? (Financial, spiritual, material, psychological, shelter, etc.)

6.4 Can Aba act on these choices? If so how? If not, why not?

6.5 What do you think will be the reaction of Aba’s husband when he finds out Aba has gone for help from an institution or organization? Why?

6.6 Is there anything Aba and the husband would like/dislike about the support given?

**PERCEPTIONS ABOUT THE ROLE OF COMBATS**

8. Have you heard or know of community members who provide support for VAW victims?

**PROBE:** Who are they? How did you get to hear of them? Have you interacted with them before? Why? Why not?

9. What kinds of assistance/support do these community members offer to VAW victims?

**PROBE:** Financial, material, shelter, psychological, spiritual, etc. How useful is this support? Why? Why not? Are the kinds of support provided by the community members acceptable? Why? Why not?

10. Personally how do you feel about community members who mediate VAW cases, and also provide support to victims?

**PROBE:** Is it something that you tolerate/allow? Why? Are you comfortable calling on them for help? Why? Why not?

11. What suggestions do you have for community members who assist with VAW case referrals and also provide support to victims?

**PROBE**: Mind their own business? Why do you say that?
